# Peptidomic Analysis of the Brain and Corpora Cardiaca-Corpora Allata Complex in the *Bombyx mori*

**DOI:** 10.1155/2012/640359

**Published:** 2012-12-17

**Authors:** Xiaoguang Liu, Xia Ning, Yan Zhang, Wenfeng Chen, Zhangwu Zhao, Qingwen Zhang

**Affiliations:** Department of Entomology, College of Agriculture and Biotechnology, China Agricultural University, China

## Abstract

The silkworm, *Bombyx mori*, is an important economic insect for silk production. However, many of the mature peptides relevant to its various life stages remain unknown. Using RP-HPLC, MALDI-TOF MS, and previously identified peptides from *B. mori* and other insects in the transcriptome database, we created peptide profiles showing a total of 6 ion masses that could be assigned to peptides in eggs, including one previously unidentified peptide. A further 49 peptides were assigned to larval brains. 17 new mature peptides were identified in isolated masses. 39 peptides were found in pupal brains with 8 unidentified peptides. 48 were found in adult brains with 12 unidentified peptides. These new unidentified peptides showed highly significant matches in all MS analysis. These matches were then searched against the National Center for Biotechnology Information (NCBI) database to provide new annotations for these mature peptides. In total, 59 mature peptides in 19 categories were found in the brains of silkworms at the larval, pupal, and adult stages. These results demonstrate that peptidomic variation across different developmental stages can be dramatic. Moreover, the corpora cardiaca-corpora allata (CC-CA) complex was examined during the fifth larval instar. A total of 41 ion masses were assigned to peptides.

## 1. Introduction

Insect neuropeptides regulate behaviors during growth, development, metamorphosis, and many other physiological processes, acting as neurohormones and neuromodulators [1]. Many *B. mori* neuropeptides have been purified and their amino acid sequences have been determined. These include adipokinetic hormone (AKH), subesophageal ganglion neuropeptides (SGNPs), corazonin, prothoracicostatic peptide (PTSP), B-myosuppressin (BMS), FMRFamide-related peptides (BRFas), and short neuropeptide F peptides (sNPFs) [2–7]. The cDNA precursors of some *B. mori* peptides, such as allatostatins A (AST-A), allatotropin (AT), and allatostatin C (AST-C), have been cloned [8–10]. The genome draft sequence in *B. mori* has been completed and this may facilitate the identification of new peptides in *B. mori* [11]. Recently, using homology searches and cDNA cloning, many new peptide genes have been annotated in this insect species, and their peptide precursor sequences have become available [12]. 

Neuropeptides have been broadly studied in many insect species, such as* Locusta migratoria*, *Apis mellifera*, and *Manduca sexta* [16, 17]. However, most peptide studies focus on a specific developmental stage, either at larval or adult, which limits appreciation of the peptidomic variations that take place across different growth stages. The aim of the present study is to profile peptide complements in eggs and in the brains of silkworms at larval, pupal, and adult stages. 

## 2. Materials and Methods

### 2.1. Insects

Silkworms from strain P50 (Dazao) were obtained from the Institute of Sericulture in Jiangsu province. They were reared on mulberry leaves at 26°C at 80% relative humidity and a 16L:8D photoperiod. For the experiments, eggs from day 3 were analyzed, and brains on day 4-5 of larvae, day 3 of pupae, and day 3 of male and female adults were separately dissected and analyzed.

### 2.2. Tissue Extraction and Liquid Chromatography

One hundred eggs were collected and incorporated into a sample, and three thus independent biological replicates (samples) were separately collected and analyzed. Similarly, one hundred of larval brains, one hundred of pupal brains, and one hundred of adult brains of *B. mori*, as well as one hundred pairs of the corpora cardiaca-corpora allata (CC-CA) complex from the fifth instar larvae, were dissected and incorporated into an independent sample, respectively, and three thus independent biological replicates were also collected and analyzed, respectively. All operations were performed on ice. Each sample was placed in a tube containing ice-cold extraction medium (methanol: water: acetic acid, 90 : 9 : 1, v/v/v), homogenized, and centrifuged at 12,000 ×g at 4°C for 15 minutes. The pellet was re extracted twice. All supernatants were pooled, the organic solvent was evaporated by vacuum centrifugation, and the residue was dissolved in 0.1% (v/v) trifluoroacetic acid (TFA). 

The samples were fractionated on an Agilent 1100 HPLC system (Agilent, USA) using a ZORBAX StableBond C18 column (4.6 mm × 250 mm, 5 *μ*m, 300 Å; Agilent, USA). The column was first eluted with 3% acetonitrile in 0.1% trifluoroacetic acid (TFA) for 10 minutes. Then acetonitrile was increased to 21% over 10 minutes. This was followed by a linear gradient of 21–60% acetonitrile/0.1% TFA over 30 minutes at a flow rate of 1 mL/min. Fractions were collected manually every minute and concentrated by the vacuum desiccator (LNG-T88, Huamei Biochemical Company, P.R.China) to about 5 *μ*L for mass spectrometry analysis.

### 2.3. MALDI-TOF Mass Spectrometry

MALDI-TOF MS analysis was performed on an autoflex II TOF/TOF instrument (Bruker Daltonics, Germany). The matrix used in the analysis was a saturated solution of recrystallized *α*-cyano-4-hydroxycinnamic acid (CHCA; Bruker Daltonics) dissolved in 70% acetonitrile containing 0.1% (v/v) TFA. Samples of 0.5 *μ*L were added to the MALDI plate, followed by 0.5 *μ*L of matrix solution. They were mixed and left to dry at room temperature. The spectra were obtained using an accelerating voltage of 19 KV in the reflection mode with a mass range m/z 700–3000. Laser power was adjusted to provide optimal signal-to-noise ratio. The measured monoisotopic masses [M + H]^+^ were compared to the calculated values of known or predicted peptides. Masses were calculated using Protein Prospector (University of California, San Francisco, CA, U.S.) [1]. MALDI-TOF-TOF mass spectra were acquired on an autoflex II TOF/TOF instrument (Bruker Daltonics, Germany). Ion fragmentation data were analyzed using FlexAnalysis software (version 3.0) from Bruker Daltonics. The mature peptide in *B. mori *from our MS/MS results were identified and confirmed by either previous publications [3–6, 8, 12–15], or the EST from the NCBI database (http://www.ncbi.nlm.nih.gov/) and silkworm database (http://silkworm.genomics.org.cn/) [18], or the NeuroPred tool SignalP 3.0 (http://www.cbs.dtu.dk/services/SignalP/).

## 3. Results

### 3.1. Peptidomics in Different Organs and Developmental Stages of *B. Mori*

In this study, over 100 ion peaks were obtained by MS profiling from analysis of eggs and all postembryonic stages. The mature peptides were evaluated by comparison of measured monoisotopic masses [M+H]^+^ against combination of bioinformatics and identified peptides in Lepidopteran insects and the predicted peptides from genome databases, such as calculated masses, score, queries matched, and sequence coverage. In total, 19 new-found and 40 previously reported mature brain peptides were identified in *B*. *mori.*

#### 3.1.1. Peptides in Eggs

Only six peptides, AKH1(Q^1^), AKH2(Q^1^), AKH3(Q^1^), CAPA-PVK-2, allatostatin-5, and *α*-SGNP, were detected in eggs. Of these, the new-found CAPA-PVK-2 is a mature peptide (Table 1 marked with star). 

#### 3.1.2. Peptides in Larval Brains

A total of 49 sequences were assigned to peptides in larval brains with 17 of them unidentified mature peptides (Table 1 marked with star). These peptides mainly include AKHs (AKH1(Q^1^), AKH2(Q^1^), and AKH3(Q^1^)), allatostatin A (allatostatin-1, -2, -4, -5, -7, -8, -8(Q^1^)), bommo-AT, bommoAST-C (AST, AST(Q^1^)), BRFa (BRFa-1, -2, -3, -4), BMS (BMS, BMS(Q^1^)), CAPA/CAP2b (CAPA-PVK-1, CAPA-PVK-2, CAPA-PK, CAPA-PVK-2(Q^1^)), CCAP, corazonin (corazonin(Q^1^)), diapause hormone (DH), leucokinin (leucokinin-2, -3), the mature peptides of the NPLP-1 precursor (AYLamide, LLHamide, NSYamide, SAMamide, and YRMamide), orcokinin (orcokinin-3, -4), PTST (PTST-3, -5, -6), SGNP (*α*-SGNP, *β*-SGNP, *γ*-SGNP), sNPF (sNPF-1, -2, -3), SIFamide, sulfakinin, and tachykinin (tachykinin-3, -4, 5, and -6), in which CAPA-PVK-1, CAPA-PVK-2, CAPA-PK, CAPA-PVK-2(Q^1^), leucokinin-2, -3, AYLamide, LLHamide, NSYamide, SAMamide, and YRMamide, SIFamid(SIF), sulfakinin, and tachykinin-3, -4, -5, and -6 are new-found mature peptides. More mature peptides were detected at this developmental stage than any other stage. 

#### 3.1.3. Peptides in Pupal Brains

39 ion masses were assigned to peptides in pupal brains, in which 8 of them are unidentified peptides in *B. mori* (Table 1 marked with star). These peptides mainly include AKHs (AKH1(Q^1^), AKH2(Q^1^), AKH3(Q^1^)), allatostatin A (allatostatin-3, -4, -5, -8, -8(Q^1^)), bommo-AT, BRFa (BRFa-1, 2, 3), B-myosuppressin (BMS, BM S (Q^1^)), CCAP, corazonin, diapause hormone (DH), the mature peptides of the NPLP-1 precursor (LLHamide, NIAALARNGLLH-NH_2_; NSYamide, NIATLAKNGYLRNSGANSY-NH_2_), Orcokinin (Orcokinin-4, -5), PTST (PTST-1, -2, -3, -4, -5, -6), SGNP(*α*-SGNP, *β*-SGNP, *γ*-SGNP), sNPF (sNPF-1, -2, -3), SIFamide, sulfakinin, and tachykinin (tachykinin-1, -3, -4). Of these, LLHamide, NSYamide, orcokinin-5, SIFamid(SIF), sulfakinin, tachykinin-1, tachykinin-3, and tachykinin-4 are new-found and unidentified mature peptides.

#### 3.1.4. Peptides in Adult Brains

48 ion masses were assigned to peptides in adult brains with 12 unidentified new peptides in *B. mori* (Table 1 marked with star). These peptides mainly include AKHs (AKH1(Q^1^), AKH2(Q^1^), AKH3, AKH3(Q^1^)), allatostatin A (allatostatin-3, -4, -5, -7, -8, -8(Q^1^)), Bommo-AT, BRFa (BRFa-1, -2, 3, -4), B-myosuppressin (BMS, BMS(Q^1^)), CAPA/CAP2b (CAPA-PVK-1, CAPA-PVK-2(Q^1^)), CCAP, corazonin (corazonin, corazonin(Q^1^)), diapause hormone (DH), NPLP-1 (AYLamide, SALGPENDYAVLKDFEDNAYL-NH_2_; LLHamide, NIAALARNGLLH-NH_2_, NSYamide, NIATLAKNGYLRNSGANSY-NH_2_), Orcokinin (Orcokinin-3, -4, -5), PTST (PTST-1, -2, -4, -5, -6), SGNP (*α*-SGNP, *β*-SGNP, *γ*-SGNP), sNPF (sNPF-1, -2, -3), SIFamide, sulfakinin, and tachykinin (tachykinin-1, -3, -4, -5, -6). Of these, CAPA-PVK-1, CAPA-PVK-2(Q^1^), LLHamide, NSYamide, orcokinin-5, SIFamide, sulfakinin, tachykinin-1, -3, -4, -5, and -6 are unidentified mature peptides.

#### 3.1.5. Peptides in the CC-CA Complex of 5th-Instar Larvae

CC-CA complexes sampled on days 4-5 of the fifth larval instar were analyzed for peptide profiles. A total of 41 ion peaks in these CC-CA complexes were assigned to peptides listed in Table 2. These peptides mainly include AKHs (AKH1(Q^1^), AKH2(Q^1^), AKH3(Q^1^)), allatostatin A (allatostatin-2, -3, -4, -5, -6, -8, -8(Q^1^)), bommo-AT, bommoAST-C (AST(Q^1^)), BRFa (BRFa-1, -2, -3, -4), B-myosuppressin (BMS, BMS (Q^1^)), CAPA/CAP2b (CAPA-PVK-1, CAPA-PVK-2, CAPA-PVK-2(Q^1^)), CCAP, corazonin (corazonin, corazonin(Q^1^)), NPLP-1 (LLHamide, NSYamide), orcokinin (orcokinin-5), PTST (PTST-3, -5, -6), SGNP (*α*-SGNP, *β*-SGNP, *γ*-SGNP), sNPF (sNPF-1, -2, -3), sulfakinin, and tachykinin (tachykinin-1, -3, -4, -6). Of these, CAPA-PVK-1, CAPA-PVK-2, CAPA-PVK-2(Q^1^), LLHamide, NSYamide, orcokinin-5, sulfakinin, tachykinin-1, -3, -4, and -6 are unidenfied mature peptides.

**Figure 1:**
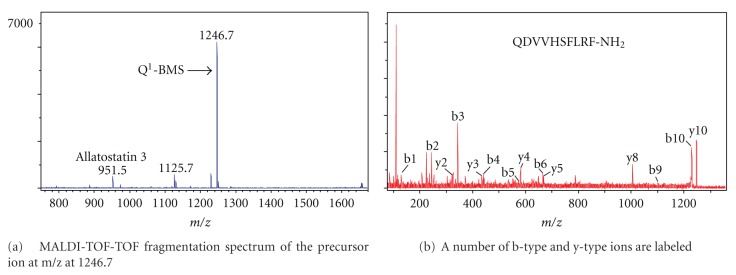
MALDI-TOF mass spectrum from separate fraction 30 of CC-CA in the fifth instar of *B. mori. *

**Figure 2:**
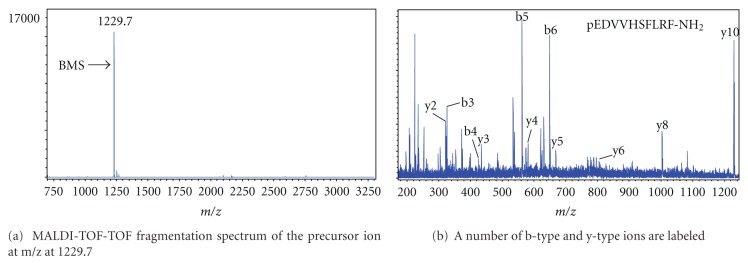
MALDI-TOF mass spectrum from separate fraction 30 of CC-CA in the fifth instar of *B. mori. *

**Figure 3:**
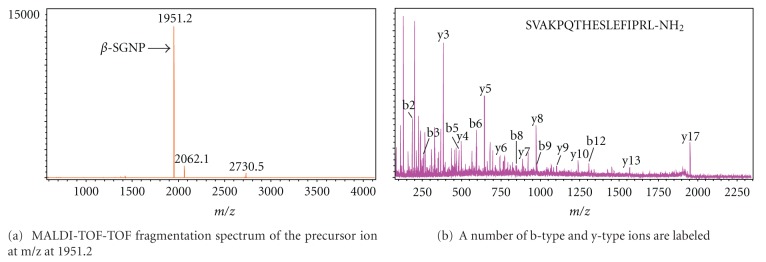
MALDI-TOF mass spectrum from separate fraction 29 of CC-CA in the fifth instar of *B. mori. *

Both the MALDI-TOF mass spectra of HPLC separation fractions from different developmental stages of silkworm brains and CC-CA extracts of the fifth instar larvae and the MALDI-TOF-TOF fragmentation spectra of the precursor ion at m/z were analyzed and identified. A number of b-type and y-type ions were labeled (Figures 1–3). Some of them are pGluat of the N-terminal.

## 4. Discussion

Peptides in insects are very important to regulate many physiological activities involved in feeding, ecdysis and metamorphosis, reproduction, energy homeostasis, circadian rhythm, anxiety, seizure, contraction of muscle, learning and memory, and so on [19–22]. Especially, variation of peptidomics at different developmental stages is huge, in which each different stage has characteristic peptides. In this study, we mapped peptides from important neuroendocrine organs, the CC-CA complex, and the brains of silkworms at different stages of growth. The aim of this study was exploring peptidomic composition at different stages because different development stages express specific physiological requirements.

Our results demonstrate that peptidomic variations during different developmental stages are profound in silkworms. In eggs, only few mature peptides were detected; we infer that developmental regulation is operates on a different physiological basis during the egg stage. ACP and AKHs are involved in mobilization of lipids and carbohydrates from fat bodies and ovaries [23]. Besides, allatostatin is involved in inhibition of JH and SGNP in diapause [24]. Compared to larval brains, pupal and adult brains lacked 3 categories (CAPA/CAP2b, NPLP-1, and Leucokinin) and 2 categories (NPLP-1, and Leucokinin), respectively. Of these 3 categories, the CAPA/CAP2b peptides have cardioacceleratory properties and increased heart rate [25–27]. The NPLP-1 was found to play a role in ecdysis behavior in *D. melanogaster* [1, 28, 29], and leucokinin is a neurohormone that participates in the regulation of water and ion homeostasis, especially the control of ion transport in the stellate cells of the insect's Malpighian tubules [1]. 

The peptidomic variation across organs between the brain and the CC-CA complex in larvae was also different. Compared to larval brains, CC-CA complex lacked 3 categories (diapause hormone, SIFamide and leucokinin). The SIFamide has been found to be responsible for courtship. Four SIFamidergic neurons and arborizations play an important function in the neuronal circuitry controlling sexual behavior in *Drosophila* [28, 29]. In addition, SIFamide may play a role in processing or transmitting tactile, olfactory, and visual stimuli, which is also important for courtship behavior and partner selection [30]. But the function of SIFamide in *B. mori* and other moths still maintains unclear. 

Nowadays, SIFamide, tachykinin (TK), CCAP, CAPA-PVK, sulfakinin, and neuropeptide-like precursor 1 (NPLP 1) were predicted by the *B. mori* genomic database [12]. MIP is another Lepidoptera peptide previously identified from *Manduca sexta* [31]. It was found to have the similar peptide precursor named as *B. mori* PTST [2–7]. Many neuropeptide precursors undergo a series of enzymatic processes, causing the production of mature, bioactive amidated neuropeptides. Each mature peptide may display a different potential function on the cellular level [32]. The AT/AST has functions in stimulating/inhibiting JH synthesis [33, 34]. The sNPF and tachykinins regulate food intake and consumption but sulfakinin inhibits food intake [35–37]. Corazonin may be the key factor in the formation of colors during the larval stage or reduction of spinning in the pupal stage [38, 39]. The orcokinins, BRFas, and PTSPs are all involved in the regulation of insect development in ecdysteroid biosynthesis, in which orcokinins have a clear prothoracicotropic activity, in contrast with BRFas [14]. While PTSPs inhibit ecdysteroid biosynthesis in the PG [15]. Again, our results indicate that peptidomics can vary greatly between different organs and developmental stages. The present study adds valuable information to the knowledge of neuropeptidomes.

In this study, all HPLC fractions were collected and analyzed by MALDI-TOF MS, and high-intensity signal peaks consistent with *B. mori* neuropeptides were fragmented by TOF-TOF for peptide identification. However, the apparatus has a limited ability to hit and break ions by TOF-TOF, which is weaker than the ion-trap mass spectrum for some peptides to be not fragmented.

## Figures and Tables

**Table 1:** The mature peptides of *Bombyx mori* from different developmental stages identified or assigned by MALDI-TOF MS.

Number	Peptide clusters	Maturae peptide name	Amino acid sequence	Reference	Transcripts	Monoisotopic masses M+H]^+^		Egg	Larva	Pupa	Adult
						Calculated	Measured				
1	AKH	AKH1(Q^1^)	QLTFTSSWG-NH_2_	[3, 13]	AB298930	1025.48	1025.58	+	+	+	+
		AKH2(Q^1^)	QLTFTPGWGQ-NH_2_	[13]	AB298931	1133.55	1133.78	+	+	+	+
		AKH3(Q^1^)	QITFSRDWSG-NH_2_	[13]	AB298938	1195.56	1194.85	+	+	+	+
		AKH3	pEITFSRDWSG-NH_2_	[13]		1179.60	1179.61				+

2	Allatostatin A	Allatostatin-1	SPQYDFGL-NH_2_	[9]	AF309090	925.42	925.57		+		
		Allatostatin-2	AYSYVSEYKRLPVYNFGL-NH_2_	[9]		2168.09	2167.621		+		
		Allatostatin-3	SRPYLFGL-NH_2_	[9]		951.52	951.41			+	+
		Allatostatin-4	ARPYSFGL-NH_2_	[9]		909.47	909.32		+	+	+
		Allatostatin-5	ARMYSFGL-NH_2_	[9]		943.46	943.46	+	+	+	+
		Allatostatin-6	ARSYSFGL-NH_2_	[9]		899.45	899.52			+	+
		Allatostatin-7	LSSKFNFGL-NH_2_	[9]		1011.54	1011.54		+		+
		Allatostatin-8(Q^1^)	QRDMHRFSFGL-NH_2_	[9]		1392.67	1393.88		+	+	+
		Allatostatin-8	pERDMHRFSFGL-NH_2_	[9]		1375.7	1375.60		+	+	+

3	Bommo-AT	Bommo-AT	GFKNVEMMTARGF-NH_2_	[8] confirmed in this study	AY970687	1486.71	1486.69		+	+	+

4	Bommo AST-C	AST-C	pEVRFRQCYFNPISCF-NH_2_	[12] confirmed in this study	AB330418	1888.90	1888.70		+		
		AST-C(Q^1^)	QVRFRQCYFNPISCF-NH_2_	[12] confirmed in this study		1906.90	1960.96		+		

5	BRFa	BRFa-1	SAIDRSMIRF-NH_2_	[5] confirmed in this study	AB234100	1194.62	1194.85		+	+	+
		BRFa-2	SASFVRF-NH_2_	[5] confirmed in this study		812.42	812.42		+	+	+
		BRFa-3	DPSFIRF-NH_2_	[5]		880.44	880.48		+	+	+
		BRFa-4	ARNHFIRL-NH_2_	[5] confirmed in this study		1025.59	1025.63		+		+

6	B-myosuppressin (BMS)	BMS	pEDVVHSFLRF-NH_2_	[6]	NM_001173411	1229.6	1229.54		+	+	+
		BMS(Q^1^)	QDVVHSFLRF-NH_2_	[6]		1246.65	1246.55		+	+	+

7	CAPA/CAP2b	CAPA-PVK-1^*⋇*^	PDGVLNLYPFPRV-NH_2 _		AB362227	1485.80	1486.54		+		+
		CAPA-PVK-2^*⋇*^	pELYAFPRV-NH_2_			975.50	975.71	+	+		
		CAPA-PK^*⋇*^	LKNGDDDVVNQNED-NH_2_			1573.69	1573.90		+		
		CAPA-PVK-2(Q^1^)^*⋇*^	QLYAFPRV-NH_2_			992.54	992.80		+		+

8	CCAP	CCAP	PFCNAFTGC-NH_2_	[12]	AB298937	958.37	956.42		+	+	+

9	Corazonin	Corazonin	pETFQYSRGWTN-NH_2_		AB106876	1369.7	1369.57			+	+
		Corazonin(Q^1^)	QTFQYSRGWTN-NH_2_			1386.63	1385.74		+		+

10	Diapause Hormone (DH)	DH	GALWFGPRL-NH_2_	[4]	D28810	1015.56	1015.73		+	+	+

11	Leucokinin	Leucokinin-2^*⋇*^	VRFSPWG-NH_2_		AB298928	847.43	847.53		+		
		Leucokinin-3^*⋇*^	KVKFSAWG-NH_2_			921.51	921.67		+		

12	NPLP-1	NPLP-1(AYL)^*⋇*^	SALGPENDYAVLKDFEDNAYL-NH_2_		AB362222	2343.09	2344.94		+		+
		NPLP-1(LLH)^*⋇*^	NIAALARNGLLH-NH_2_			1261.73	1261.57		+	+	+
		NPLP-1(NSY)^*⋇*^	NIATLAKNGYLRNSGANSY-NH_2_			2026.02	2027.19		+	+	+
		NPLP-1(SAM)^*⋇*^	NLASIARLRSYSAM-NH_2 _			1551.82	1551.95		+		
		NPLP-1(YRM)^*⋇*^	NIQALARDGYRM-NH_2_			1406.70	1406.72		+		

13	Orcokinin	Orcokinin-3(I)	NFDEIDRSSLNTFV-NH_2_	[14]	AB298932	1655.78	1656.74		+		+
		Orcokinin-4(II)	NFDEIDRSSMPFPYAI-NH_2_	[14]		1900.87	1901.98		+	+	+
		Orcokinin-5	YRPDYPMDEIDLSHFPVGS-NH_2_	Confirmed in this study		2237.01	2237.39			+	+

14	PTST	PTST-1	AWQDLNSAW-NH_2_	[6]	AB073553	1089.49	1089.74			+	+
		PTST-2	GWQDLNSAW-NH_2_	[15]		1075.47	1075.50			+	+
		PTST-3	APEKWAAFHGSWG-NH_2_	[15]		1442.67	1441.83		+	+	
		PTST-4	GWNDISSVWG-NH_2_	[15]		1119.50	1120.93			+	+
		PTST-5	AWQDMSSAW-NH_2_	[15]		1080.43	1080.67		+	+	+
		PTST-6	AWSALHGTW-NH_2_	[15]		1027.49	1027.67		+	+	+

15	SGNP	*α*-SGNP	IIFTPKL-NH_2_	[4]	D28810	830.53	830.78	+	+	+	+
		*β*-SGNP	SVAKPQTHESLEFIPRL-NH_2_	[4]		1951.05	1951.20		+	+	+
		*γ*-SGNP	TMSFSPRL-NH_2_	[4]		937.47	937.67		+	+	+

16	sNPF	sNPF-1	SPSRRLRF-NH_2_	[10]	AB330419	1017.58	1017.45		+	+	+
		sNPF-2	TPVRLRF-NH_2_	[10]		887.53	887.72		+	+	+
		sNPF3	APSMRLRF-NH_2 _	[10]		976.53	976.74		+	+	+

17	SIFamide	SIFamide^*⋇*^	TYRKPPFNGSIF-NH_2_		AB298923	1425.74	1425.60		+	+	+

18	Sulfakinin	Sulfakinin^*⋇*^	GDDTFDDYGHLRF-NH_2_		AB362223	1556.65	1556.92		+	+	+

19	Tachykinin	Tachykinin-1^*⋇*^	IPQGFLGMR-NH_2_		AB298929	1017.54	1017.60			+	+
		Tachykinin-3^*⋇*^	APLGFTGVR-NH_2_			916.51	916.50		+	+	+
		Tachykinin-4^*⋇*^	AANMHQFYGVR-NH_2_			1292.61	1292.48		+	+	+
		Tachykinin-5^*⋇*^	PYDLSIRGKFIGVR-NH_2_			1619.91	1619.03		+		+
		Tachykinin-6^*⋇*^	GQMGFFGMR-NH_2_			1029.45	1029.74		+		+

Masses were calculated using ProteinProspector (University of California, San Francisco, CA, USA). ^*⋇*^Newly characterized peptides in this study.

**Table 2:** The mature peptides of *Bombyx mori* from larvae CC-CA identified or assigned by MALDI-TOF MS.

Number	Peptide clusters	Maturae peptide name	Amino acid sequence	Reference	Transcripts	Monoisotopic masses M+H]^+^		Larvae CC-CA
						Calculated	Measured
1	AKH	AKH1(Q^1^)	QLTFTSSWG-NH_2_	[3, 13]	AB298930	1025.48	1025.58	+
		AKH2(Q^1^)	QLTFTPGWGQ-NH_2_	[13]	AB298931	1133.55	1133.78	+
		AKH3(Q^1^)	QITFSRDWSG-NH_2_	[13]	AB298938	1195.56	1194.85	+

2	Allatostatin A	Allatostatin-2	AYSYVSEYKRLPVYNFGL -NH_2_	[9]	AF309090	2168.09	2167.621	+
		Allatostatin-3	SRPYLFGL-NH_2_	[9]		951.52	951.41	+
		Allatostatin-4	ARPYSFGL-NH_2_	[9]		909.47	909.32	+
		Allatostatin-5	ARMYSFGL-NH_2_	[9]		943.46	943.46	+
		Allatostatin-6	ARSYSFGL-NH_2_	[9]		899.45	899.52	+
		Allatostatin-8(Q^1^)	QRDMHRFSFGL-NH_2_	[9]		1392.67	1393.88	+
		Allatostatin-8	pERDMHRFSFGL-NH_2_	[9]		1375.7	1375.60	+

3	Bommo-AT	Bommo-AT	GFKNVEMMTARGF-NH_2_	[4]	AY970687	1486.71	1486.69	+

4	Bommo AST-C	AST-C(Q^1^)	QVRFRQCYFNPISCF-NH_2_	[12]	AB330418	1906.90	1960.96	+

5	BRFa	BRFa-1	SAIDRSMIRF-NH_2_	[5]	AB234100	1194.62	1194.85	+
		BRFa-2	SASFVRF-NH_2_	[5]		812.42	812.42	+
		BRFa-3	DPSFIRF-NH_2_	[5]		880.44	880.48	+
		BRFa-4	ARNHFIRL-NH_2_	[5]		1025.59	1025.63	+

6	B-myosuppressin (BMS)	BMS	pEDVVHSFLRF-NH_2_	[6]	NM_001173411	1229.6	1229.54	+
		BMS(Q^1^)	QDVVHSFLRF-NH_2_	Confirmed in this study		1246.65	1246.55	+

7	CAPA/CAP2b	CAPA-PVK-1^*⋇*^	PDGVLNLYPFPRV-NH_2_		AB362227	1485.80	1486.54	+
		CAPA-PVK-2^*⋇*^	pELYAFPRV-NH_2_			975.50	975.71	+
		CAPA-PVK-2(Q^1^)^*⋇*^	QLYAFPRV-NH_2_			992.54	992.80	+

8	CCAP	CCAP	PFCNAFTGC-NH_2_		AB298937	958.37	956.42	+

9	Corazonin	Corazonin	pETFQYSRGWTN-NH_2_	Confirmed in this study	AB106876	1369.7	1369.57	+
		Corazonin(Q^1^)	QTFQYSRGWTN-NH_2_	Confirmed in this study		1386.63	1385.74	+

10	NPLP-1	NPLP-1(LLH)^*⋇*^	NIAALARNGLLH-NH_2_		AB362222	1261.73	1261.57	+
		NPLP-1(NSY)^*⋇*^	NIATLAKNGYLRNSGANSY-NH_2_			2026.02	2027.19	+

11	Orcokinin	Orcokinin-5	YRPDYPMDEIDLSHFPVGS-NH_2_	Confirmed in this study	AB298932	2237.01	2237.39	+

12	PTST	PTST-3	APEKWAAFHGSWG-NH_2_	[15]	AB073553	1442.67	1441.83	+
		PTST-5	AWQDMSSAW-NH_2_	[15]		1080.43	1080.67	+
		PTST-6	AWSALHGTW-NH_2_	[15]		1027.49	1027.67	+

13	SGNP	*α*-SGNP	IIFTPKL-NH_2_	[4]	D28810	830.53	830.78	+
		*β*-SGNP	SVAKPQTHESLEFIPRL-NH_2_	[4]		1951.05	1951.32	+
		*γ*-SGNP	TMSFSPRL-NH_2_	[4]		937.47	937.67	+

14	sNPF	sNPF-1	SPSRRLRF-NH_2_	[10]	AB330419	1017.58	1017.45	+
		sNPF-2	TPVRLRF-NH_2_	[10]		887.53	887.72	+
		sNPF3	APSMRLRF-NH_2_	[10]		976.53	976.74	+

15	Sulfakinin	Sulfakinin^*⋇*^	GDDTFDDYGHLRF-NH_2_		AB362223	1556.65	1556.92	+

16	Tachykinin	Tachykinin-1^*⋇*^	IPQGFLGMR-NH_2_		AB298929	1017.54	1017.60	+
		Tachykinin-3^*⋇*^	APLGFTGVR-NH_2_			916.51	916.50	+
		Tachykinin-4^*⋇*^	AANMHQFYGVR-NH_2_			1292.61	1292.48	+
		Tachykinin-6^*⋇*^	GQMGFFGMR-NH_2_			1029.45	1029.74	+

Masses were calculated using ProteinProspector (University of California, San Francisco, CA, USA). The mature peptide marked. ^*⋇*^Newly characterized peptides in this study.
